# Health- and Age-Related Workplace Factors as Predictors of Preferred, Expected, and Actual Retirement Timing: Findings from a Swedish Cohort Study

**DOI:** 10.3390/ijerph18052746

**Published:** 2021-03-08

**Authors:** Marta Sousa-Ribeiro, Claudia Bernhard-Oettel, Magnus Sverke, Hugo Westerlund

**Affiliations:** Department of Psychology, Stockholm University, 10691 Stockholm, Sweden; claudia.bernhard.oettel@su.se (C.B.-O.); magnus.sverke@psychology.su.se (M.S.); hugo.westerlund@su.se (H.W.)

**Keywords:** retirement process, older workers, aging in the workplace, extended working lives

## Abstract

To address the challenges of demographic aging, governments and organizations encourage extended working lives. This study investigates how individual health- and age-related workplace factors contribute to preferred, expected and actual retirement timing, as well as to the congruency between preferences vs. expectations, and preferences vs. actual retirement. We used data from a representative Swedish longitudinal sample comprising 4058 workers aged 50–64, with follow-up data regarding actual retirement timing available for 1164 respondents. Multinomial logistic regression analyses suggest that later preferred, expected, and actual retirement timing were, to different extent, influenced by better health, an age-friendly workplace and feeling positive regarding the future at work. Emotional exhaustion, age-related inequalities at work and experiencing aging as an obstacle increased the likelihood of preferring to retire earlier than one expected to, over retiring at the time one expected to. Those with better health and positive work prospects were less likely to prefer retiring earlier than they expected to, and more likely to being “pulled toward working until 65 and beyond”, compared to being “pulled toward early retirement”. Experiencing aging as an obstacle decreased the chances of being “pulled toward working until 65 and beyond”. The results provide insights on how to facilitate extended working lives.

## 1. Introduction

Many countries are facing the issue of an aged society [1]. While increasing numbers of active and healthy older people is a remarkable achievement [2], the fact that people on average receive retirement pensions for a longer period than ever before pressures the pension and social security systems, and raises concerns regarding the adequacy of the pension system to prevent old-age poverty in the future [1,3].

Understanding the factors that hinder or stimulate longer working lives is therefore relevant for developing effective policies and interventions to increase the employability of older workers and encourage later retirement [4,5]. A wealth of studies has identified multiple factors involved in retirement decisions [6,7,8,9,10,11]. Most research has focused on either retirement timing preferences, or intentions or actual retirement timing. Less often, these indicators have been considered in repeated study designs following older workers longitudinally. In addition, the congruency between the individual’s preferred and expected, as well as between preferred and actual retirement timing have received fairly little attention. Moreover, there are still relatively few studies using large and representative samples and longitudinal data [7].

Wang and Shultz [8] clustered the various factors involved in the retirement process into four categories: individual, family, job and organizational, and socioeconomic. Some of the most relevant predictors of retirement decisions are at the individual level [8], in particular economic and health-related factors [10]. The role of health in older workers’ retirement is however not straightforward [7]. Furthermore, retirement studies have neglected psychological health [12]. Apart from the individual, the workplace has a central role in shaping the opportunities for working longer [13], but not enough attention has been paid to the role of the organizational context in older workers’ retirement decision making [5].

Addressing these voids, the present study investigates the relative contribution of health (self-rated health and emotional exhaustion), psychological age climate at the workplace (perceived age-related inequalities and valorization of older workers’ experience), and perceived future at work (experiencing aging as a future obstacle at work, positive work prospects and opportunities to work after retirement age) to retirement decision making, in a large representative longitudinal sample of older workers in Sweden. We focused specifically on (1) preferred retirement timing, (2) expected retirement timing, (3) actual retirement timing, (4) the congruency between preferred and expected retirement timing, and (5) the congruency between preferred and actual retirement timing. We develop hypotheses that are theoretically and empirically grounded for investigating aims 1–4. However, regarding aim 5, research questions are framed instead, given the lack of earlier evidence.

### 1.1. The Swedish Context

Sweden is an interesting context to study retirement decision making, considering the significant influence workers have in deciding their retirement timing, at least at a formal level [14]. In Sweden, there is no mandatory retirement age, and individuals may draw an earnings-related pension from the age of 62, with economic incentives to postpone retirement, while the minimum eligible age to a supplementary guarantee pension (a basic protection for those who have no or low lifetime earnings) is 65 years. [15]. Moreover, an employee is protected by the law of employment security until the age of 68, and there is no maximum age for starting taking up a pension [15]. The pension can be drawn as 25–100 percent of the whole, and it is possible for the individual to continue in paid employment and earning new pension entitlements after starting to draw the pension [16]. Furthermore, the retiree is allowed to suspend and subsequently resume pension payments at any time [16]. Sweden has a relatively high employment rate of workers aged 60–64 (70%), but this rate sharply decreases among those aged 65–69 (24%) [1]. The average age to start taking up the retirement pension was 64.6 years in 2019 and, among those who turned 65 years in 2019, around 40 percent started drawing their pension at 65 [15]. While 65 is still regarded as the “normal” retirement age, this is about to change, as an increasing number of people retire at different ages, both earlier and later than 65 [15]. Notwithstanding the relatively late-exit culture compared to other countries [17], preferences for early retirement seem prevalent in Sweden. In a survey conducted in 2015 [3], the average age until which people thought they would be able to do their current job or a similar one was 68.0 for men and 67.1 for women, whereas the average age up to which people wanted to work was 63.3 and 62.8, respectively. Thus, people still want to retire much earlier than they probably can and will.

### 1.2. Retirement Decision-Making Process—Preferred, Expected, and Actual Retirement Timing

There exists no single, distinct, and universal definition of retirement status [18] and there are divergences in the literature with regard to its empirical operationalization, which may be based in subjective or objective assessments, or a combination of the two [19]. Wang and Shi [9] defined retirement as “an individual’s exit from the workforce, which accompanies decreased psychological commitment to and behavioral withdrawal from work” (p. 211). In line with this definition, retirement can be both a psychological process in conceptualization and a life status in empirical operationalization. In the present study, retirement is conceptualized as a decision-making process and empirically operationalized as a labor market status in which the individual receives a retirement/pension income and has left the workforce. Retirement timing, in turn, refers to the age or relative point at which the individual retires [7].

Retirement has been described as a decision-making process occurring over a period of time, starting with the preference to retire (thinking about retirement), continuing to the decision to retire (intention), and ending with actual retirement [20,21]. One key dimension here concerns the individual’s preferred retirement timing, i.e., the optimal age at which the individual would like to retire “when intrinsic consideration is more extremely decisive, and […] when financial consequences in case of retirement need not be considered” [4] (p. 19). Preferred retirement timing may be an indicator of the individual’s commitment to the labor force [22], and is “a better measure of individuals’ non-coercively structured taste for retirement” [4] (p. 149) than expected retirement timing. This refers to the age at which the individual realistically expects to retire “when extrinsic work and retirement alternatives (such as, e.g., eligibility age and financial opportunities) are likely considered” [4] (p. 19). Expected retirement timing may be a proxy for the planned or intended retirement timing. Factors playing a role in retirement preferences may differ from those having an impact on intentions to retire and on actual retirement [11]. The three indicators considered in the present study—preferred, expected and actual retirement timing—may therefore provide complementary information on how different individual and workplace-related factors contribute to different facets in the retirement decision-making process.

Conceptualizing retirement as a decision-making process assumes that retirement decisions reflect a motivated choice [9]. However, not all retirement decisions are voluntary and without constraints [23] and, irrespective of the individual’s preferences, individual and contextual factors may facilitate or restrict the control workers have over their retirement [24]. According to the push/pull model of retirement [25], these factors can be push forces out of work (acting as constraints to work) or pull forces toward retirement (acting as incentives to retirement). The push/pull perspective may also be applied to investigate decisions for working longer [26], either because the individual is “pushed” to continue working due to constraints to retirement, such as the need for an income, or “pulled” toward working longer, for instance because he or she is attached to the working role and feels attracted by certain job features.

The difference between preferred and expected retirement timing creates a motivational (mis)match reflected in three conditions: (1) “potential early retirees”, representing workers who prefer to retire before the expected age; (2) workers who prefer to retire when they expect to do so, and (3) “workers disposed to late retirement”, denoting those who prefer to retire later than they realistically expect to [27]. With increasing retirement ages, the proportion of those who expect, and eventually will need, to work longer than preferred will probably grow over the coming decades [23], and more knowledge is needed about factors that may influence how preferred retirement timing is associated with expected retirement timing at a given time [28]. With few exceptions [4,27] this seems not to have been considered in retirement research and is therefore examined in the present study.

Furthermore, few studies have longitudinally investigated the congruency between preferred retirement timing at one point in time and subsequent actual retirement. Steiber and Kohli’s [29] study is one exception, which, however, took a retrospective approach, as preferred retirement timing was measured asking already retired individuals at what age they would have liked to retire. This may not be accurate, as participants are required to recall their former preference, which may have been adapted to the actual retirement timing [14] or be influenced by the experience of retirement. In another study, Solem et al. [30], used a prospective measure of retirement preference, but investigated only a limited number of predictive factors, mostly of sociodemographic nature, for preferred and actual retirement timing. Further longitudinal research using a prospective measure of preferred retirement timing and investigating other influential factors is therefore needed. Filling this gap, our study investigates how health- and age-related workplace factors are associated with different types of (in)congruencies between preferred and actual retirement timing.

Instead of examining the discrepancy between preferred and actual retirement timing quantitatively (i.e., difference in number of years), we adopted an alternative approach and investigated different combinations of preferences and actual retirement, based on the relative point of retirement timing (at age 64 or earlier, at age 65 or later) for preferred and actual retirement. By doing so, we focused on the quality of the congruency, rather than only on its direction (i.e., actual retirement earlier than preferred, at the preferred timing, or later than preferred) and magnitude. Using this categorization, we were able to investigate four categories of (in)congruencies between preferred and actual retirement timing: (1) those who were “pulled toward early retirement” (had a preference for early retirement, i.e., at 64 or earlier, and actually retired at 64 or earlier); (2) those who were “pushed to continue working” (had a preference for early retirement, but actually retired at a later timing than preferred, i.e., at age 65 or later); (3) those who were “pushed out of work” (had a preference for retiring at age 65 or later, but actually retired earlier than preferred, i.e., at 64 or earlier), and; (4) those who were “pulled toward working until 65 and beyond” (had a preference for retiring, and actually retired, at age 65 or later).

### 1.3. Factors Associated with the Retirement Process

This study focuses on the contribution of health (self-rated health and emotional exhaustion), psychological age climate at the workplace (perceived age-related inequalities and valorization of older workers’ experience), and perceived future at work (experiencing aging as a future obstacle at work, positive work prospects and opportunities to work after retirement age) to the retirement decision-making process.

#### 1.3.1. Health

Health is a core individual resource which affects individuals’ work ability and the likelihood of a longer working life [31]. The role of health in retirement decision making may be discussed in the light of the conservation of resources (COR) theory [32,33]. According to the COR theory, individuals are driven to maintain and protect their current resources, and those with fewer resources are more exposed to further resource losses and less capable of acquiring new ones. Along this line, older workers with poorer health may perceive themselves as more vulnerable and less resourceful, as well as having poorer work ability, and may consider work a threat to one’s remaining health. Under these circumstances, early retirement may be a way to protect health from further declines, the so-called “health protection” exit pathway [34].

Poor health has consistently been associated with early retirement [7,10], while good health status is one of the strongest predictors of late retirement preferences and decisions [35,36]. Some studies have however not found a significant association between poor health and expected retirement timing [37], early retirement intention [38] or actual early retirement [39]. This may be because good health in some cases is related to early retirement, particularly among those who can afford to retire—the ‘maximization of life’ exit pathway [34], counterbalancing the most typically found association between poor health and labor market exit.

Poor psychological health also contributes to labor market withdrawal, and its impact may be even more pervasive than poor physical health [40]. However, most research has focused on individuals’ general state of health and few longitudinal studies have investigated the role of psychological health in retirement [12,41]. Indeed, in their literature review on the influence of different health measures on three labor market exit pathways (unemployment, disability pension and early retirement), van Rijn et al. [42] found no studies on the association between mental health and early retirement. In a meta-analysis on the antecedents of early retirement [10], only 27 percent of the studies included some indicator of mental health. This meta-analysis found a relatively weak effect size for the relationship between poor mental health and early retirement (r_c_ = 0.20).

In Warr’s [43] perspective of psychological well-being, burnout is an indicator of job-related affect, a domain-specific form of well-being. It is therefore a relevant factor to include in a study on retirement. Burnout is “a psychological syndrome in response to chronic interpersonal stressors on the job” [44] (p. 399) with three dimensions: depersonalization, lack of accomplishment, and emotional exhaustion. The latter refers to feeling overextended and emotionally and physically depleted of one’s resources and is the principal component and basic individual stress dimension of burnout [44]. Emotional exhaustion decreases both the willingness and ability to work until the age of 65 [45] and is associated with a stronger intention to leave the workforce [12].

Considering this background, we derive the following hypotheses (H) and research questions (RQ):

H1: Better self-rated health increases the likelihood of later (a) preferred, (b) expected, and (c) actual retirement timing, while it (d) decreases the likelihood of preferring to retire earlier than one expects to.

RQ1: How does self-rated health relate to different types of (in)congruencies between preferred and actual retirement timing?

H2: Emotional exhaustion decreases the likelihood of later (a) preferred, (b) expected, and (c) actual retirement timing, while it (d) increases the likelihood of preferring to retire earlier than one expects to.

RQ2: How does emotional exhaustion relate to different types of (in)congruencies between preferred and actual retirement timing?

#### 1.3.2. Psychological Age Climate in the Workplace

According to the model of successful aging [46], optimal aging refers to “aging under development-enhancing and age-friendly environmental conditions” (p. 8). The individual (rather than collective) perceptions concerning the age-friendliness of a work environment is named psychological age climate [47]. In the present study, psychological age climate in the workplace comprises two aspects: perceived age-related inequalities and perceived valorization of older workers’ experience at the workplace.

Regarding the first aspect, perceived age-related inequalities or discrimination at work is a recognized push factor out of work, e.g., [48], as early retirement may be a way to cope with the stigmatization of aging in the workplace [49]. Based on Swedish studies, Isaksson et al. [50] indeed considered ageism as an obstacle to extended working lives. In contrast, policies and procedures for equal treatment of employees from different age groups appear to promote successful aging [51], and the perception that age is not used in the organization as a criterion for distinguishing between workers has been found to be positively related to a higher value placed on work [49].

The valorization of older workers’ experience at the workplace comprises the second psychological age climate aspect considered in this study. An age-friendly workplace is one where older workers are regarded as a valued resource and are respected within the organization [52], and organizations valuing older workers’ experience and knowledge have been found to be more successful in encouraging older workers to remain in employment [53]. Armstrong-Stassen [54] indeed found that “recognition and respect” was a particularly important human resources management (HRM) practice in encouraging retirees’ decision to remain in—or return to—the workforce. In contrast, Soidre [55] found that men who felt undervalued at work preferred early retirement. Based on this background, we derive the following hypotheses and research questions:

H3: Perceived age-related inequalities at the workplace decreases the likelihood of later (a) preferred, (b) expected, and (c) actual retirement timing, while it (d) increases the likelihood of preferring to retire earlier than one expects to.

RQ3: How do perceived age-related inequalities at the workplace relate to different types of (in)congruencies between preferred and actual retirement timing?

H4: Perceived valorization of older workers’ experience at the workplace increases the likelihood of later (a) preferred, (b) expected, and (c) actual retirement timing, while it (d) decreases the likelihood of preferring to retire earlier than one expects to.

RQ4: How does the perceived valorization of older workers’ experience at the workplace relate to different types of (in)congruencies between preferred and actual retirement timing?

#### 1.3.3. Perceived Future at Work

Older workers’ opportunities at work are an absolute condition for a longer working life [31] and the individual perceptions of one’s future time in the work context (occupational future time perspective) have been found to influence the career development of older workers [56]. In the present study, we investigate how three such factors—experiencing aging as a future obstacle at work, positive work prospects, and perceived opportunity for continuing working—relate to older workers’ retirement decision making.

Concerning the first, older workers who experience ageist attitudes in the workplace may develop age-related anxiety, internalize negative age-related stereotypes [57], such as age-related declines, and perceive aging as an obstacle for their future at work. This may result in a reduced willingness to continue working, as part of a self-fulfilling prophecy [58], hampering the individual’s potential for active aging and increasing the willingness to retire [49,59]. The assumption of negative consequences resulting from aging has moreover been associated with lower subjective well-being [60], which may also threaten the permanence in the workforce.

Second, accompanying the profound changes in labor-market structures and pensions systems, particularly in the last two decades, the traditional unidirectional and linear career paths have gradually changed to more diverse and dynamic patterns [61] and two new paradigms (lifetime employability [62] and lifelong career development [63]) have emerged. Accordingly, retirement has been conceptualized as a late career development stage, which assumes the continued potential for growth and renewal of late careers [9]. In line with this, Van Solinge and Henkens [5] found that perceived growth opportunities at work contributed to actual late retirement. Data from the European Working Conditions Survey suggest, in turn, that poor career prospects contribute to unsustainability of work and are a key determinant for older workers leaving the workforce, while having good career prospects is a pull factor into employment [64].

Third, while active aging policies for extending working lives rely on the assumption that the decision to remain in the organization is at disposal of the older workers, this is not always the case [65]. Organizational retirement practices and norms may suggest to older workers when it is time to leave and may, therefore, influence retirement intentions and actual retirement timing [5,7]. In Topa et al.’s [10] meta-analysis, workplace timing of retirement was indeed found to be the best predictor of early retirement. In contrast, opportunities to work at later ages within the organization have been found to contribute to longer working lives [66].

Against this background, we derive the following hypotheses and research questions:

H5: Experiencing aging as a future obstacle at work decreases the likelihood of later (a) preferred, (b) expected, and (c) actual retirement timing, while it (d) increases the likelihood of preferring to retire earlier than one expects to.

RQ5: How do the experiences of aging as a future obstacle at work relate to different types of (in)congruencies between preferred and actual retirement timing?

H6: Feeling positive regarding one’s future work prospects increases the likelihood of later (a) preferred, (b) expected, and (c) actual retirement timing, while it (d) decreases the likelihood of preferring to retire earlier than one expects to.

RQ6: How do positive future work prospects relate to different types of (in)congruencies between preferred and actual retirement timing?

H7: Perceived opportunities to work in the organization after retirement age increases the likelihood of later (a) preferred, (b) expected, and (c) actual retirement timing, while it (d) decreases the likelihood of preferring to retire earlier than one expects to.

RQ7: How do perceived opportunities to work after retirement age relate to different types of (in)congruencies between preferred and actual retirement timing?

## 2. Materials and Methods

### 2.1. Sample and Procedure

The study sample was drawn from the Swedish Longitudinal Occupational Survey of Health (SLOSH), a longitudinal cohort survey originally representative of the Swedish working population, which focus on associations between work organization, work environment and health [67]. All participants are followed biennially since 2006 via postal questionnaires. The survey has two versions, one for individuals working for at least 30 percent of full-time (including self-employment) and another for individuals working less than 30 percent or who are temporarily or permanently outside the workforce, including retirees. The Regional Research Ethics Board in Stockholm has approved both SLOSH (ref no. 2012/272-2/5) and the present study (ref no. 2017/1720-31/5).

This study is based on data from 4548 respondents to the questionnaire for those in paid work (including self-employment) in 2010 (T1), aged 50–64 years. After excluding 490 participants with missing data on relevant variables, the valid sample at T1 comprised 4058 individuals with a mean age of 57 years (SD = 4). Of these, 35 percent were married/cohabiting women, 21 percent were single/not cohabiting women, 29 percent were married/cohabiting men, and 15 percent were single/not cohabiting men. The majority (63%) were white-collar workers.

In 2016 (T2), 988 respondents (24%) had dropped out from the study. A total of 1895 (47%) answered to the questionnaire for those in paid work (of which 167 were aged 66 years or older) and 1175 (29%) answered to the questionnaire for those non-working (of which 1027 reported being retired, receiving some type of earnings-related old-age pension, and not receiving disability pension nor any type of sickness benefit). Among the 1027 participants who reported being retired in 2016, 30 respondents did not indicate the year at which they left paid work and were therefore excluded from the T2 sample because their actual retirement timing could not be determined. Among the remaining 997 participants, 443 (44%) had retired at age 64 or earlier, 338 (34%) at the age of 65, and 216 (22%) at age 66 or later. The 167 participants who were working in 2016 and aged 66 years or older were included in the latter group (as they would necessarily retire at age 66 or later), such that this group comprised a total of 383 respondents. Altogether, the sample at T2 comprised 1164 respondents.

Dropout analyses compared respondents participating in both waves (*n* = 3070) with those who responded at T1 but not at T2 (*n* = 988). Results from these analyses showed that compared to those who participated in both waves, dropouts included slightly younger employees (0.46 years) (*t* = 2.96, *p* < 0.01) and more individuals in the lowest income level (*χ*2 = 30.79, *p* < 0.001), more individuals in blue-collar occupations (*χ*2 = 48.97, *p* < 0.001), and more single women and men (*χ*2 = 17.02, *p* < 0.01). Further, dropouts reported slightly poorer self-rated health (mean difference = 0.13) (*t* = 4.24, *p* < 0.001).

### 2.2. Measures

Table 1 presents the descriptive statistics and bivariate correlations for all study variables.

#### 2.2.1. Dependent Variables

Single items were used to measure preferred retirement timing [“At what age do you want to retire (as things look today)?”] and expected retirement timing [“At what age do you expect to retire (as things look today)?”], both assessed at T1. The responses were given as full years, which were later coded into three categories (1 = at age 64 or earlier; 2 = at age 65; 3 = at age 66 or later) for analytic purposes. This categorization has been used in other studies on retirement preferences, e.g., [55].

The congruency between preferred and expected retirement timing was computed by subtracting the expected retirement age from preferred retirement age at T1. The resulting values (negative values, values equalizing zero and positive values) were recoded into 3 categories (1 = preferred to retire earlier than one expected to; 2 = preferred to retire at the time one expected to; 3 = preferred to retire later than one expected to).

Actual retirement timing was collected at T2. For participants who responded to the questionnaire for those not working, reported were receiving a retirement pension, and indicated the year they left paid work, actual retirement timing was computed subtracting the year of birth from the year they reported to have left paid work. The resulting variable was coded in three categories: (1 = at age 64 or earlier; 2 = at age 65; 3 = at age 66 or later) (the latter including those still in paid work who were aged 66 or older).

The variable congruency between preferred and actual retirement timing was based on the combination of the variables preferred retirement timing at T1 and actual retirement timing collected at T2. We first isolated the category “at age 64 or earlier” and merged the categories “at age 65” and “at age 66 or later” for each variable. The four resulting combinations of preferred vs. actual retirement timing were then: 1 = pulled toward early retirement (those who preferred to and actually retired at age 64 or earlier), 2 = pushed to continue working (those who remained involuntarily at work, i.e., preferred to retire at age 64 or earlier but retired at age 65 or later), 3 = pushed out of work (those who preferred to retire at age 65 or later but retired at age 64 or earlier); 4 = pulled toward working until 65 and beyond (those who preferred to and actually retired at age 65 or later).

#### 2.2.2. Independent Variables

All independent variables were measured at T1.

##### Health

Self-rated health was assessed with a single item (“How would you rate your general state of health?”) scored on a 5-point rating scale, ranging from (1) “very good” to (5) “very poor”. The response scale was reversed such that a high value reflects better subjective health. Emotional exhaustion was measured with the 5-item exhaustion subscale of the Maslach Burnout Inventory-General Survey [68]. All items were scored on a 6-point rating scale, ranging from (1) “everyday” to (6) “sometimes per year or less/never.” A sample item is “My job makes me feel emotionally drained”. The response scale was reversed such that high scores correspond to high levels of emotional exhaustion. The Cronbach’s alpha for this scale was 0.89.

##### Psychological Age Climate in the Workplace

Single items were used to measure age-related inequalities at the workplace (“Have you noticed any inequalities how older and younger workers are treated at your workplace?”) and valorization of older workers’ experience at the workplace (“Are elderly workers’ experience appreciated at your workplace?”). These single items are part of the Nordic Questionnaire for Monitoring the Age Diverse Workforce (QPSNordic-ADW) [69] and were scored in a 5-point response scale ranging from (1) “very seldom/never” to (5) “very often/all the time” and (1) “not at all” to (5) “very much”, respectively.

##### Perceived Future at Work

Single items were also used to measure aging as a future obstacle at work (“Do you believe that the fact that you are getting older will cause you some problems at your work in the future?”) and positive work prospects (“Do you feel positive about how your work will develop in the future?), which were drawn from the Nordic Questionnaire for Monitoring the Age Diverse Workforce QPSNordic-ADW [69] and scored in a 5-point response scale ranging from (1) “not at all” to (5) “very much”. Opportunities to work after retirement age was assessed by a single item (“Are there opportunities at your job to work, either full or part-time, after the normal retirement age?”), with responses coded (0) “no” and (1) “yes”.

#### 2.2.3. Covariates

All covariates were assessed at T1. SLOSH contains information obtained from register data about age (based on year of birth), gender (0 = man, 1 = woman), marital status (0 = not married/cohabiting, 1 = married/cohabiting), income (yearly income in thousands of Swedish crowns [SEK], recoded into a variable based on percentiles: 1 = low [up to 278,000 SEK], 2 = medium [279,000–359,000 SEK], and 3 = high incomes [360,000 SEK and more]), and occupational status (coded 0 = white-collar workers and 1 = blue-collar workers, based on the SSYK [Swedish Standard Classification of Occupations], which is closely related to ISCO [International Standard Classification of Occupations]). These variables have been found to relate to retirement preferences and behavior, e.g., [7] and were therefore included in this study as covariates. Given the significant impact of domestic context on late-career and retirement decisions [70,71], an interaction term between gender and marital status (1 = not-cohabiting women, 2 = married/cohabiting women, 3 = not-cohabiting men, 4 = married/cohabiting men) was used instead of investigating each of these variables separately.

### 2.3. Data Analyses

Multivariate multinomial logistic regression analyses were calculated using SPSS version 26 to investigate the associations of the independent variables and the covariates with the outcome variables. The odds ratios were estimated for each independent variable, fully adjusted for the other variables, relative to a reference category: “at age 64 or earlier” in predicting preferred, expected and actual retirement timing, “preferred to retire at the time one expected to” in predicting the congruency between preferred and expected retirement timing, and “pulled toward early retirement” in the prediction of the congruency between preferred and actual retirement timing. Collinearity diagnostics indicated that multicollinearity was not a concern.

## 3. Results

### 3.1. Descriptive Results

Descriptive statistics (not reported in any table) showed that most participants (57%) preferred to retire at age 64 or earlier, while 36 percent preferred to retire at age 65, and only 7 percent reported a preference to retire at age 66 or later. As concerns expected retirement timing, most participants (58%) expected to retire at age 65, 28 percent expected to retire at age 64 or earlier and 14 percent expected to retire at age 66 or later. Looking at the actual retirement timing, 38 percent of participants included in the T2 sample had retired at age 64 or earlier, 29 percent at age 65, and 33 percent retired at age 66 or later. In relation to the congruency between preferred and expected retirement timing, while most respondents preferred to retire at the time they expected to (53%), the proportion of “potential early retirees” (those participants who preferred to retire earlier than they expected to) was considerably large (44%), and only 3 percent expressed the preference for retiring later than they expected to. Finally, with regard to the congruency between preferred and actual retirement timing, 31 percent of participants in the T2 sample were “pulled toward early retirement”, 18 percent were “pushed to continue working” 8 percent were “pushed out of work”, and 44 percent were “pulled toward working until 65 and beyond”.

### 3.2. Results From the Multinominal Regression Analyses

The results of the multivariate multinomial logistic regression analyses are presented in Table 2 (predictions of preferred, expected, and actual retirement timing), and Table 3 (predictions of the congruency between preferred and expected retirement timing and the congruency between preferred and actual retirement timing).

#### 3.2.1. Health

In line with H1a, better self-rated health increased the likelihood of preferring to retire at age 66 or later relative to preferring to retire at age 64 or earlier. Self-rated health was, however, not significantly related to the odds of preferring to retire at age 65 over preferring to retire at 64 or earlier. The findings did not support H1b, as self-rated health was not significantly related to the odds of expecting to retire at later ages over expecting to retire at age 64 or earlier. Regarding actual retirement, H1c was partially supported, as those with better self-rated health were more likely to actually retiring at age 66 or later, but not at age 65, compared to retiring at age 64 or earlier. Better self-rated health was moreover related to a lower likelihood of preferring to retire earlier than one expected to, compared to preferring to retire at the time one expected to, which is in line with H1d. With regard to the congruency between preferred and actual retirement timing (RQ1), those with better health were more likely to being “pulled toward working until 65 and beyond” compared to being “pulled toward early retirement”.

Emotional exhaustion, on the other hand, decreased the likelihood of preferring to retire at age 65 and at age 66 or later compared to preferring to retire at age 64 or earlier, which is in line with H2a. Contrary to H2b, however, those with higher levels of emotional exhaustion were more likely to expect to retire at age 66 or later compared to expecting to retire at age 64 or earlier. Emotional exhaustion did not predict the likelihood of actual late retirement over early retirement, as assumed in H2c. Consistent with H2d, emotional exhaustion increased the likelihood of preferring to retire earlier than one expected to, relative to preferring to retire at the time one expected to. Regarding RQ2, higher levels of emotional exhaustion were associated with a higher probability of being “pushed to continue working” relative to being “pulled toward early retirement”.

#### 3.2.2. Psychological Age Climate in the Workplace

Perceiving age-related inequalities at the workplace did not predict later preferred, expected, and actual retirement over early retirement, and therefore H3a-c were not supported by the findings. As predicted by H3d, this variable was associated with a higher likelihood of preferring to retire earlier than one expected to over preferring to retire at the time one expected to. Furthermore, with regard to RQ3, age inequalities at work did not explain the probability of falling in any of the categories of (in)congruencies between preferred and actual retirement timing relative to being “pulled toward early retirement”. The results did not support H4a-d, as the perception that older workers’ experience was valued at the workplace did not predict later preferred, expected, and actual retirement, neither the likelihood of preferring to retire earlier than one expected to over preferring to retire at the time one expected to. Concerning RQ4, this variable was not relevant in predicting the congruency between preferred and actual retirement timing.

#### 3.2.3. Perceived Future at Work

Experiencing aging as a future obstacle at work decreased the likelihood of preferring to retire at age 65 relative to preferring to retire at age 64 or earlier, in line with H5a. This variable, however, did not predict expected or actual retirement timing, and therefore H5b-c were not supported. Moreover, experiencing aging as a future obstacle at work was associated with a higher risk of preferring to retire earlier than one expected to compared to preferring to retire at the time one expected to, in line with H5d, but also with the preference to retire later than one expected to. With respect to the congruency between preferred and actual retirement timing (RQ5), experiencing aging as a future obstacle at work decreased the odds of being “pulled toward working until 65 and beyond”, relative to being “pulled toward early retirement”.

In line with H6a-c, feeling positive regarding one’s work prospects increased the likelihood of preferring to, expecting to, and actually retiring at age 65 and at age 66 or later, relative, respectively, to preferring to, expecting to, and actually retiring at age 64 or earlier. Furthermore, consistent with H6d, positive work prospects decreased the risk of preferring to retire earlier than one expected to over preferring to retire at the time one expected to. With respect to the congruency between preferred and actual retirement timing (RQ6), feeling positive regarding one’s future work prospects was associated with a higher likelihood of being pulled toward working until 65 and beyond compared to being pulled toward early retirement.

Finally, having the opportunity to work in the organization after retirement age was associated with a higher likelihood of preferring to, and expecting to retire at age 65 and at age 66 or later, compared to retiring at age 64 or earlier. These findings give support to H7a-b. However, opportunities to work after retirement did not predict later actual retirement relative to earlier retirement, neither the probability of preferring to retire earlier than one expected to, compared to preferring to retire at the time one expected to, and therefore H7c-d were not supported. With regard to RQ7, having the opportunity to work in the organization after retirement age was not associated with the probability of falling into any of the categories of (in)congruencies between preferred and actual retirement.

#### 3.2.4. Covariates

Among the control variables, age increased the likelihood of preferring to, and actually retiring, at age 65 and at age 66 or later, compared to preferring to, and actually retiring at age 64 or earlier, respectively. Furthermore, higher age was related to a lower risk of preferring to retire earlier than one expected to, over preferring to retire at the time one expected to. Finally, age increased the odds of being “pushed to continue working”, “pushed out of work” and being “pulled toward working until 65 and beyond” compared to being “pulled toward early retirement”.

Being a married/co-habiting woman (in comparison to be a married/co-habiting man) decreased the likelihood of preferring to, expecting to, and actually retiring at age 66 or later relative, respectively, to preferring to, expecting to, and actually retiring at age 64 or earlier. Furthermore, this group had a lower probability of being “pushed to continue working” and “pulled toward working until 65 and beyond”, over being “pulled toward early retirement”. In contrast, single woman (in comparison to married/co-habiting man) had a higher likelihood of preferring to retire at age 65 over preferring to retire at age 64 or earlier, as well as expecting to retire at age 65 and at age 66 or later relative to expecting to retire at age 64 or earlier.

Regarding income, participants in the low and medium income levels (in comparison to those in the highest income level) were more likely to expect to retire at age 65 relative to expecting to retire at age 64 or earlier. People in medium income levels had moreover a higher probability of actually retiring at age 65 relative to retiring at age 64 or earlier, and of preferring to retire earlier than they expected to, over preferring to retire at the time they expected to. With respect to the associations between income and the congruency between preferred and actual retirement timing, those in the low and medium income levels (relative to those in the highest income level) were more likely to be “pushed to continue working” over being “pulled toward early retirement”, and those in the medium-income level had also a higher likelihood of being “pulled toward working until 65 and beyond”.

Blue-collar workers, relative to white-collar workers, had a lower likelihood of preferring to and actually retiring at age 66 or later, compared, respectively, to preferring to, and actually retiring at age 64 or earlier.

## 4. Discussion

Demographic population changes and an aging workforce demand for longer working lives, and policy-makers and organizations may benefit from a better understanding of factors encouraging older workers to remain in employment. This study examined the relative importance of health (self-rated health and emotional exhaustion), psychological age climate in the workplace (age-related inequalities and valorization of older workers’ experience), and perceived future at work (experiencing aging as a future obstacle at work, positive work prospects and opportunities to work after retirement age) for older workers’ preferred, expected and actual retirement timing, as well as for the congruency between preferred vs. expected retirement timing and preferred vs. actual retirement timing, in a large representative longitudinal sample of older workers in Sweden.

Participants showed a clear preference for early retirement, which is not in line with the current policy reforms toward increasing retirement ages. Moreover, most participants expected to retire at the age of 65, suggesting that expectations regarding retirement timing were largely framed by a “default” retirement age (65) that still seems to be regarded as the norm (at least in Sweden). A relatively high share of participants had a preference for retiring earlier than they expected to and, while the majority who had retired between waves did so around the preferred timing, a considerable proportion of participants actually retired later than they would have preferred, i.e., were pushed to continue working. Working longer than preferred may have negative consequences for health and well-being [29]. It may also be problematic to the organization, as those workers who would prefer to leave the workforce without actually leaving can start detaching, be “mentally distant” from work, and may be difficult to encourage [12]. Since T1 data was collected in 2010, future research may investigate the extent to which the extensive debate on the need to extend working lives in Sweden has influenced both preferred and expected retirement timing.

The factors investigated in this study contributed differently to preferred, expected and actual retirement timing, in line with earlier research, e.g., [5,11,24], as well as to the congruency between preferred vs. expected retirement timing and preferred vs. actual retirement timing. This underscores the relevance of examining these different outcomes in the same study.

### 4.1. Health

We examined the contribution of self-rated health (H1a–d) and emotional exhaustion (H2a–d) in predicting (a) preferred, (b) expected, and (c) actual retirement timing, as well as (d) the congruency between preferred and expected retirement timing. Furthermore, two research questions (RQ1 and RQ2) were framed to investigate the role of self-rated health and emotional exhaustion, respectively, in explaining the congruency between preferred and actual retirement timing.

Those with better self-rated health were more likely to prefer late retirement, over preferring to retire earlier, as expected in H1a. This finding may be discussed in the light of the COR theory [32], as those with impaired health may fear further health declines and consider that leaving the workforce would protect their health—a valued individual resource—from further declines. In line with this, research [40,57] has found that health pessimism was negatively associated with the willingness to postpone retirement. Furthermore, those with poorer health may perceive lower levels of work ability and feel less capable to deal with demands at work, preferring to leave employment earlier than those who feel more capable to perform [72].

However, contrary to the prediction in H1b, self-rated health did not predict late expected retirement timing, as also was the case in Beehr et al.’s [37] study. Participants in the present study reported—on average—a relatively good health status and even among those reporting poorer health, health issues may not have been sufficiently serious to affect their expected retirement timing. Furthermore, as noted by Nilsson et al. [73], the social insurance reform in Sweden, implemented in 2006–2010, tightened the conditions qualifying for a disability pension and this may explain why health may affect retirement preferences but not the (more) realistic expectations toward retirement timing, as those with poor health—but not eligible to a disability pension—may not have a chance to retire as early as they would prefer [29]. This argument is also supported by the finding that better self-rated health decreased the likelihood of preferring to retire earlier than one expected to over preferring to retire at the time one expected to, in line with H1d. Our longitudinal findings show that better health at T1 increased the odds of actual retirement timing at age 66 or later, over retiring at 64 or earlier. Moreover, regarding the congruency between preferred and actual retirement timing, those with better health were more likely to be “pulled toward working until 65 and beyond” compared to being “pulled toward early retirement”. These findings highlight the importance of health in facilitating and encouraging the permanence of older workers in the workforce, contributing to extended working lives.

With regard to emotional exhaustion, our findings support the assumption that feelings of exhaustion increase the desire to leave the workforce. In fact, and in line with earlier research [45] and with H2a, those experiencing higher levels of emotional exhaustion were less likely to prefer retiring at 65 and at 66 or later, compared to preferring to retire at age 64 or earlier. On the other hand, emotional exhaustion increased the odds of expecting to retire at age 66 or later over expecting to retire at 64 or earlier (contrary to H2b), and was associated with higher odds of preferring to retire earlier than one expected to, over preferring to retire at the time one expected to (as predicted by H2d). It seems, then, that participants experiencing higher levels of emotional exhaustion may face constraints to the implementation of their retirement preferences, which is suggested also by the finding that emotional exhaustion was associated with an increase in relative probability of being “pushed to continue working” (that is, to work longer than one had wished) over being “pulled to early retirement” (that is, to prefer and be able to retire early). These findings suggest that, while policies increasing the statutory retirement ages may be effective in extending working lives, they may not automatically prolong the number of productive years [12]. Emotional exhaustion has in earlier studies indeed been associated with poorer work performance and organizational commitment [74], and higher levels of sickness absence [75].

It may be that there is a reciprocal relationship between emotional exhaustion and feelings of being forced to continue working longer than preferred, which may in part be explained by perceiving a lack of opportunities to retire at the preferred timing, one of these being insufficient income. Our findings show that participants in the low and medium income levels, compared to those in the highest income level, had an increased risk of being “pushed to continue working” relative to be “pulled toward early retirement”. Earlier research [26] has shown, in turn, that the perceptions of “job lock” due to financial needs, that is, the situation in which the worker would prefer to retire but perceives one cannot not because of the need of an income, may increase the levels of emotional exhaustion. Future research may further explore these relationships.

### 4.2. Psychological Age Climate in the Workplace

This study investigated the role of two aspects of psychological age climate in the workplace—perceived age-related inequalities (H3a–d) and perceived valorization of older workers’ experience (H4a–d)—in predicting (a) preferred, (b) expected, and (c) actual retirement timing, along with (d) the congruency between preferred and expected retirement timing. Additionally, two research questions (RQ3 and RQ4) were formulated regarding the contributions of, respectively, perceived age-related inequalities and valorization of older workers’ experience to the congruency between preferred and actual retirement timing.

Perceiving inequalities between younger and older workers—a proxy of age discrimination—was not a significant predictor of preferred, expected and actual late retirement timing, and, therefore, H3a–c were not supported. Some studies have also found a lack of associations between age discrimination and intended retirement timing [76] and actual early retirement [66]. It may be that the relationship between age discrimination and retirement decisions is not direct but rather mediated by other factors, such as health or emotional exhaustion. For instance, Volpone and Avery [77] found that burnout mediated the association between perceived age discrimination and turnover intention, and future research could investigate whether this indirect relationship is also found in the prediction of retirement decisions. Perceived inequalities were negatively correlated with self-rated health and positively with emotional exhaustion in the present study, and Nilsson [36] found that the attitude of managers to older workers in the organization and perceived age discrimination were associated with self-rated health. On the other hand, the present study found that perceiving inequalities at the workplace was associated with a higher likelihood of preferring to retire earlier than one expected to over preferring to retire at the time one expected to, in line with H3d. This suggests that discriminatory workplaces may have an impact on older workers’ motivation to continue working, which may partially occur via negative work attitudes. Perceived age discrimination has indeed been negatively associated with affective organizational commitment [78], employee engagement [79] and job satisfaction [80]. With regard to RQ3, perceived age-related inequalities did not predict any type of (in)congruency between preferred and actual retirement timing (relative to being “pulled toward early retirement”) in the present study.

Surprisingly, the perception that older workers’ experience was valued at the workplace was not associated with any of the outcomes, and therefore H4a-d were not supported. It may be that the referent used in the item—‘older workers’ in general instead of the respondent (him/her)self—made it less relevant to differentiate participants based on their preferred, expected and actual retirement timing. Armstrong-Stassen and Schlosser [81], for instance, found that the extent to which older workers perceived themselves that they mattered and were a valued member of the organization was associated with their intention to remain with their organization through a sense of belonging. In the same line, De Wind et al. [82] found that employees who perceived high appreciation at work were less prone to retire. Future research may investigate the contribution of organizational-based self-esteem (OBSE)—which reflects “the self-perceived value that individuals have of themselves as important, competent, and capable within their employing organizations” [83] (p. 593)—to retirement decisions. Older workers perceiving a non-age friendly climate may have lower levels of OBSE and consequently wish to retire in an attempt to preserve their self-concept [84]. Associations have indeed been found between OBSE and employee motivation, job satisfaction, organizational commitment and performance, as well as turnover intentions and actual turnover [83]. Concerning RQ4, perceiving that older workers’ experience was valued at the workplace did not predict the likelihood of any type of (in)congruency between preferred and actual retirement timing relative to being “pulled toward early retirement”.

### 4.3. Perceived Future at Work

We investigated how experiencing aging as a future obstacle at work (H5a–d), feeling positive regarding one’s future work prospects (H6a–d), and perceiving opportunities for continuing working after retirement age (H7a–d) related to older workers’ (a) preferred, (b) expected, and (c) actual retirement timing, as well as (d) the congruency between preferred and expected retirement timing. Moreover, we examined how these factors contributed to the congruency between preferred and actual retirement timing (RQ5, RQ6 and RQ7).

The experience of aging as a future obstacle at work decreased the odds of preferring to retire at 65 over preferring to retire at 64 or earlier, which is in line with H5a. This hypothesis was however only partially supported, as experiencing aging as an obstacle was not significantly associated with the likelihood of preferring to retire at 66 or later relative to preferring to retire at age 64 or earlier. Moreover, this variable did not predict expected neither actual retirement at 65, or at 66 or later, over earlier retirement, and therefore H5b-c were not supported. With respect to the congruency between preferred and expected retirement timing, experiencing aging as a future obstacle at work increased the likelihood of both preferring to retire earlier than one expected to and preferring to retire later than one expected to, over preferring to retire at the time one expected to. For some people, perceiving that aging will be an obstacle at work may increase the willingness to retire early, for instance because of internalized negative age-related stereotypes or perceiving/anticipating an age-related decline that was not sufficiently disabling though so that they realistically expected to retire that early. For other individuals, however, aging may be perceived as an unsurpassable obstacle in the future (and perhaps already at the present) due to, for example, health-related problems or impairments in work-ability hindering individuals’ potential for working in later ages and that they expect will eventually push them out of work earlier than they would prefer. Concerning the congruency between preferred and actual retirement timing, our longitudinal findings show in turn that experiences of aging as a future obstacle at work decreased the likelihood of being “pulled toward working until 65 and beyond” in comparison to being “pulled toward early retirement”.

Feeling positive regarding one’s future work prospects was the only factor associated with all outcomes in this study, and seems an important contributor to a longer participation in the workforce. In line with previous literature [5,64] and H6a–c, this study found that having positive work prospects was positively related to the likelihood of preferring, expecting and actually retiring at age 65 and age 66 or later, compared to preferring, expecting, and actually retiring at 64 or earlier. Furthermore, the more positive one was regarding one’s future work prospects, the lower the odds of preferring to retire earlier than one expected to compared to preferring to retire at the time one expected to. With regard to the congruency between preferred and actual retirement timing, the present longitudinal findings show that positive future work prospects at T1 increased the likelihood of being “pulled toward working until 65 and beyond” relative to being “pulled toward early retirement”. While careers are now more dynamic and fluid than in the past [85], there is still a widespread belief that career development is no longer relevant to workers as they get older [86], which relies on traditional vocational development theories in which the late career period comprises a maintenance stage followed by a disengagement stage [87]. On the contrary, this study’s findings show the importance of organizations to recognize the continued potential for growth and renewal of late careers [9], in line with contemporary career development theories, e.g., [88].

Perceiving that the organization offered opportunities to work after retirement age was positively associated with the likelihood of preferring to, and expecting to retire at age 65 and at age 66 or later (over preferring and expecting to retire at 64 or earlier), in line with H7a–b. These findings suggest that the organizational openness to work after retirement may signal older employees that they are a valuable asset to the organization [71], encouraging extended working lives. However, this variable was not significantly associated with the odds of actual retirement timing at later ages relative to retiring early (H7c). This finding is consistent with previous research [5], having found that organizational early retirement culture is associated with lower (planned) retirement ages but not actual retirement age, what reinforces the assumption that factors that underlie actual retirement may differ from those contributing to retirement preferences, which are formed in the years preceding retirement.

### 4.4. Methodological Considerations

This study has a number of methodological issues that should be considered. The first concerns the cross-sectional nature of the data used in the analyses to predict preferred and expected retirement timing and the congruency between preferred and expected retirement timing, as no causal inference between the independent and dependent variables can be drawn [89]. However, the study used also longitudinal data, with a time lag of six years between measurements, to predict actual retirement timing and the congruency between preferred and actual retirement timing. The longitudinal findings, as well as previous research and theoretically and empirically grounded models of retirement timing, e.g., [7], suggest that the directions of causality proposed are more plausible than their reverse.

Second, issues regarding the generalizability of the findings should also be acknowledged. This study was conducted in Sweden, a European country with a relatively generous welfare system, where, at least at a formal level, people have a considerable decision latitude with regard to their retirement timing [14]. Steiber and Koli [29] found indeed that among 12 European countries, the share of voluntary retirees—those whose preferred retirement timing matched their actual retirement timing—was the highest in the Nordic countries, including Sweden. It would be interesting to investigate, for instance, which factors, among those investigated in the present study, are associated with each type of (in)congruency between preferred and actual retirement timing in different welfare and pension systems.

The third methodological issue concerns the exclusive use of self-report data, which may result in a source of uncontrolled measurement error due to common method variance [90]. However, this study focused on associations between individuals’ perceptions (regarding health and work) and their retirement preferences/expectations/behavior, and self-report assessments are considered adequate in such cases [91]. Furthermore, self-rated health seems a more important predictor of retirement than more objective health measures, such as diagnosed diseases [92]. The use of register data from the national pensions’ authority would allow for more objective information and homogeneous criteria to assess actual retirement timing. Nevertheless, retirement was operationalized in this study as the exit from the workforce and in this case, it is preferable to assess retirement via self-report data, since people in Sweden may be receiving a pension and continue working in some way [16].

Fourth, the use of single item measures may be considered a shortcoming of this study, which made use of SLOSH data that was not collected for the purposes of the present study. SLOSH has however the strength of being a large nationally representative cohort of people who are followed over time regardless of employment status at follow-up [67]. Moreover, the use of short or single item measures is frequent in retirement research, as well-established and reliable measures of constructs of interest are often not available in large interdisciplinary studies [7]. Furthermore, most variables examined in this study may be considered sufficiently narrow or unambiguous to fulfil the criteria for being assessed by a single item [93].

A fifth methodological question concerns potential sources of bias resulting from sample selection issues. Retirement age is not known for those who dropped out from the study between waves, and data on actual retirement timing used in the longitudinal analyses was not available for those participants who were either too young to have had the possibility to retire at T2, or had reached the possibility to retire but had not yet retired. In the latter case, we could only estimate actual retirement timing for those who were aged 66 and older, whereas for all others the retirement timing remained unknown. Furthermore, those participants with better health may be overrepresented in SLOSH, both at T1 and over time (the so-called “healthy worker effect” [94]), which may hamper the generalization of the results [67] and underestimate some of the relationships between the study variables (in particular health) and the outcomes.

### 4.5. Implications for Practice

To respond to the challenges posed by population and workforce aging, governments worldwide have increased the statutory retirement age and established benefit increments for later retirement [1,3]. Nevertheless, these measures are likely not enough for people to work longer [95]. The findings with regard to the role of health highlight the importance of organizations helping older workers to maintain their health and productivity [96]. Healthy working conditions as well as health promotion and disability management interventions at the workplace should indeed be a concern throughout the working-life span [66]. Our findings concerning the influence of the perception of aging as a future obstacle at work show the importance of adopting an age-sensitive perspective in the organization [97], by implementing HRM practices tailored at the individual that counteract age-related stereotypes, promote successful and sustainable aging in the workplace and prolong a healthy and productive working life [71,98]. Having positive work prospects was the most significant predictor of extended working lives in this study. This finding shows the importance of providing older workers with the opportunity to participate in continuous learning and career development activities and facilitating the achievement of one’s career goals within the organization [64]. Schalk and Desmette [71] warn however that human resources managers should be careful in not inducing ageism when implementing such practices, as there is the risk of precipitating age prejudice from younger colleagues and the internalization of negative age stereotypes from older workers themselves.

## 5. Conclusions

This study focused on how health- and age-related workplace factors associate with preferred, expected and actual retirement, as well as with the congruency between preferred vs. expected retirement timing and preferred vs. actual retirement timing in a large representative longitudinal sample of older workers in Sweden. The findings show a clear preference for early retirement, which is not in line with the extended working lives political agenda, and reinforce the importance of understanding factors associated with later retirement, that may inform the design of policies and practices that go beyond economic incentives to postpone retirement. Overall, better health, an age-friendly work environment and, principally, feeling positive regarding the future at work, contributed, although in different extents, to preferring, expecting and actually retiring at later ages.

An important contribution of this study concerns the congruency between preferred and expected retirement timing, specifically, finding that a great share of participants preferred to retire earlier than they expected to. These individuals, at a particular moment, may feel that they are at risk of “job lock”, that is, of remaining involuntarily at the workforce, because they expect that they will not be able to retire as early as they wished, what may have negative consequences for both the individual and the organization over time. A better understanding of factors that contribute to older workers preferring to retire earlier than they expect to be able to, and which factors may decrease such a preference, may provide inputs for the design of interventions targeted at promoting well-being and positive work attitudes among older workers. The present study found that higher levels of emotional exhaustion, perceiving age-related inequalities at the workplace and experiencing aging as a future obstacle at work, contributed to a higher likelihood of preferring to retire earlier than one expected to, over preferring to retire at the time one expected to, while better self-rated health and feeling positive regarding one’s work prospects decreased the odds of preferring to retire earlier than one expected to.

Few studies have longitudinally investigated the correspondence between preferred and actual retirement timing and this is another relevant contribution of this study. Better self-rated health and positive work prospects increased the likelihood of being “pulled toward working until 65 and beyond” over being “pulled toward early retirement”, while experiencing aging as a future obstacle at work decreased the likelihood of preferring to and actually working until 65 and beyond. Those reporting higher levels of emotional exhaustion, in turn, were more likely to remain involuntarily in work, that is, to be pushed to continue working relative to being pulled toward early retirement. The study findings provide insights for policy-makers and organizations wishing to encourage their older employees to extend their working life.

## Figures and Tables

**Table 1:** Descriptive statistics and bivariate correlations between the variables under study ^a^.

		Range	M	SD	1	2	3	4	5	6	7	8	9	10	11	12	13	14	15
	**Dependent Variables**																		
1	Preferred retirement timing ^b^	1–3	1.51	0.63															
2	Expected retirement timing ^b^	1–3	1.86	0.63	0.54 *														
3	Actual retirement timing ^b^	1–3	1.95	0.84	0.52 *	0.53 *													
	**Health**																		
4	Good self-rated health	1–5	3.99	0.80	0.13 *	0.03 *	0.09 *												
5	Emotional exhaustion	1–6	2.19	1.15	−0.15 *	−0.00	−0.03	−0.41 *											
	**Psychological age climate**								‘‘‘										
6	Age-related inequalities at the workplace	1–5	2.08	1.02	−0.08 *	−0.01	−0.04	−0.15 *	0.24 *										
7	Valorization of older workers’ experience	1–5	3.41	0.98	0.09 *	0.02	0.10 *	0.20 *	−0.26 *	−0.38 *									
	**Perceived future at work**																		
8	Aging as a future obstacle at work	1–5	2.42	1.04	−0.13 *	−0.03	−0.10 *	−0.27 *	0.29 *	0.24 *	−0.26 *								
9	Positive work prospects	1–5	2.93	1.06	0.17 *	0.07 *	0.17 *	0.24 *	−0.35 *	−0.21 *	0.39 *	−0.25 *							
10	Opportunities work after retirement age	0–1	0.67	0.47	0.10 *	0.09 *	0.10 *	0.07 *	−0.03	−0.04 *	0.17 *	−0.06 *	0.10 *						
	**Covariates**																		
11	Age	50–64	56.99	4.20	0.20 *	−0.02	0.39 *	0.01	−0.01	0.01	−0.01	−0.08 *	−0.02	0.06 *					
12	Gender (woman)	0–1	0.56	0.50	−0.03	−0.01	−0.06 *	0.03	0.14	0.02	0.01	0.05 *	−0.01	0.05 *	−0.02				
13	Marital status (married/cohabiting)	0–1	0.65	0.48	−0.03 *	−0.11 *	−0.09 *	0.06 *	−0.04	−0.02	0.04 *	−0.04 *	0.05 *	0.03	0.09 *	−0.03 *			
14	Income group ^c^	1–3	2.00	0.81	0.05 *	−0.01	0.04	0.11 *	−0.08	0.03 *	0.07 *	−0.12 *	0.10 *	0.01	−0.00	−0.32 *	0.05 *		
15	Occupational status (blue-collar)	0–1	0.63	0.48	−0.04 *	0.01	−0.05	−0.06 *	−0.04	−0.06 *	−0.04 *	0.12 *	−0.06 *	−0.07 *	−0.03	−0.11 *	−0.07 *	−0.38 *	

Note: ^a^ all variables measured at T1 (2010), N = 4058, with the exception of actual retirement timing, assessed at T2 (2016), N = 1164; ^b^ 1 = at 64 or earlier, 2 = at 65, 3 = at 66 or later; ^c^ 1 = low, 2 = medium, 3 = high; * *p* < 0.05.

**Table 2:** Estimates from multivariate multinomial logistic regression models predicting preferred and expected retirement timing at T1 (N = 4058) and actual retirement timing at T2 (N = 1164).

	Preferred Retirement Timing ^a^				Expected Retirement Timing ^a^				Actual Retirement Timing ^a^			
	Age 65 *n* = 1456		Age 66 or Later *n* = 301		Age 65 *n* = 2364		Age 66 or Later *n* = 572		Age 65 *n* = 338		Age 66 or Later *n* = 383	
	OR	95% CI	OR	95% CI	OR	95% CI	OR	95% CI	OR	95% CI	OR	95% CI
**Health**												
Better self-reported health	1.07	0.97–1.18	**1.45**	**1.20–1.76**	1.06	0.95–1.17	1.15	0.99–1.33	1.14	0.91–1.43	**1.29**	**1.03–1.62**
Emotional exhaustion	**0.86**	**0.80–0.93**	**0.84**	**0.72–0.97**	0.99	0.92–1.06	**1.15**	**1.03–1.27**	1.16	0.99–1.37	1.16	0.99–1.37
**Psychological age climate at the workplace**												
Age-related inequalities at the workplace	0.97	0.90–1.05	0.91	0.79–1.04	1.02	0.94–1.01	1.02	0.91–1.14	0.90	0.76–1.06	0.96	0.81–1.13
Valorization of older workers’ experience	0.98	0.91–1.07	0.94	0.81–1.10	1.00	0.92–1.09	0.94	0.83–1.07	0.85	0.70–1.02	1.02	0.85–1.23
**Future at work**												
Aging as a future obstacle at work	**0.92**	**0.85–0.98**	0.91	0.79–1.05	0.93	0.86–1.00	0.95	0.85–1.06	0.85	0.72–1.00	0.93	0.79–1.09
Positive work prospects	**1.21**	**1.12–1.30**	**1.55**	**1.34–1.78**	**1.09**	**1.01–1.17**	**1.31**	**1.17–1.46**	**1.33**	**1.13–1.57**	**1.47**	**1.24–1.73**
Opportunities to work after retirement age ^b^	**1.27**	**1.10–1.47**	**1.83**	**1.35–2.48**	**1.23**	**1.05–1.43**	**2.03**	**1.60–2.57**	1.09	0.77–1.53	1.29	0.91–1.83
**Covariates**												
Age	**1.09**	**1.08–1.11**	**1.15**	**1.12–1.19**	0.99	0.98–1.01	0.98	0.96–1.01	**1.74**	**1.58–1.91**	**1.77**	**1.60–1.94**
Married/cohabiting women ^c^	1.01	0.84–1.22	**0.50**	**0.36–0.71**	0.91	0.75–1.11	**0.48**	**0.36–0.64**	0.75	0.50–1.12	**0.45**	**0.30–0.67**
Single woman ^c^	**1.47**	**1.20–1.81**	1.28	0.86–1.75	**1.88**	**1.49–2.39**	**1.87**	**1.37–2.54**	1.60	0.97–2.64	1.26	0.77–2.06
Single man ^c^	1.12	0.90–1.39	1.01	0.68–1.49	1.01	0.80–1.27	1.23	0.90–1.67	0.94	0.54–1.63	1.05	0.63–1.76
Low annual income ^d^	0.96	0.79–1.17	0.94	0.65–1.35	**1.34**	**1.09–1.66**	1.16	0.86–1.56	1.41	0.90–2.21	1.13	0.72–1.76
Medium annual income ^d^	1.07	0.90–1.28	0.92	0.67–1.26	**1.57**	**1.30–1.89**	1.26	0.97–1.63	**1.62**	**1.09–2.43**	1.33	0.90–1.96
Blue-collar workers ^e^	1.06	0.90–1.24	**0.65**	**0.48–0.89**	1.17	0.99–1.39	0.95	0.74–1.21	0.86	0.59–1.26	**0.64**	**0.44–0.94**
**Chi-square (df)**	440.65 *** (28)				219.67 *** (28)				332.53 *** (28)			
**Nagelkerke R^2^**	0.12				0.06				0.28			

Note: Reference categories: ^a^ “age 64 or earlier”; ^b^ “no”; ^c^ “married/cohabiting men”; ^d^ “high annual income”; ^e^ “white-collar workers”; significant results are highlighted in **bold**; *** *p* ≤ 0.001.

**Table 3:** Estimates from multivariate multinomial logistic regression models predicting the congruency between preferred and expected retirement timing at T1 (N = 4058), and the congruency between preferred and actual retirement timing at T1-T2 (N = 1164).

	Congruency Between Preferred and Expected Retirement Timing ^a^				Congruency Between Preferred and Actual Retirement Timing ^b^					
	Preferred Retiring Earlier Than Expected *n* = 1765		Preferred Retiring Later Than Expected *n* = 140		Pushed to Continue Working *n* = 207		Pushed Out of Work *n* = 87		Pulled Toward Working Until 65 and Beyond *n* = 514	
	OR	95% CI	OR	95% CI	OR	95% CI	OR	95% CI	OR	95% CI
**Health**										
Better self-reported health	**0.84**	**0.76–0.92**	0.99	0.78–1.28	1.23	0.95–1.59	1.30	0.91–1.85	**1.33**	**1.05–1.67**
Emotional exhaustion	**1.17**	**1.10–1.26**	1.03	0.86–1.23	**1.35**	**1.13–1.61**	1.04	0.81–1.34	1.06	0.89–1.25
**Psychological age climate at the workplace**										
Age-related inequalities at the workplace	**1.10**	**1.02–1.18**	1.14	0.95–1.37	1.00	0.83–1.22	1.01	0.78–1.30	0.89	0.75–1.06
Valorization of older workers’ experience	1.01	0.93–1.10	0.90	0.73–1.10	0.99	0.80–1.23	1.00	0.75–1.34	0.90	0.74–1.10
**Future at work**										
Aging as a future obstacle at work	**1.12**	**1.04–1.20**	**1.26**	**1.05–1.51**	0.91	0.75–1.09	0.82	0.64–1.06	**0.82**	**0.69–0.97**
Positive work prospects	**0.84**	**0.78–0.91**	1.09	0.90–1.31	1.21	1.00–1.47	1.24	0.96–1.60	**1.64**	**1.38–1.95**
Opportunities to work after retirement age ^c^	0.94	0.81–1.09	0.86	0.59–1.23	1.24	0.83–1.85	1.28	0.75–2.18	1.25	0.88–1.78
**Covariates**										
Age	**0.86**	**0.85–0.88**	0.96	0.92–1.00	**1.51**	**1.36–1.69**	**1.20**	**1.05–1.36**	**2.04**	**1.84–2.26**
Married/cohabiting woman ^d^	0.87	0.72–1.04	0.82	0.51–1.31	**0.52**	**0.32–0.83**	0.68	0.37–1.26	**0.53**	**0.35–0.80**
Single woman ^d^	1.02	0.83–1.25	0.91	0.54–1.53	1.05	0.58–1.90	1.03	0.47–2.28	1.68	1.00–2.83
Single man ^d^	0.93	0.75–1.16	1.00	0.58–1.73	0.74	0.39–1.39	0.45	0.17–1.18	0.93	0.54–1.59
Low annual income ^e^	1.21	0.99–1.47	1.16	0.70–1.91	**1.86**	**1.09–3.15**	1.88	0.93–3.80	1.27	0.80–2.01
Medium annual income ^e^	**1.29**	**1.09–1.54**	1.17	0.76–1.82	**1.82**	**1.14–2.91**	1.69	0.90–3.21	**1.54**	**1.02–2.31**
Blue-collar workers ^f^	1.03	0.88–1.21	0.73	0.48–1.10	0.79	0.51–1.23	1.24	0.70–2.17	0.77	0.52–1.14
**Chi-square (df)**	544.11 *** (28)				408.85 *** (42)					
**Nagelkerke R^2^**	0.16				0.32					

Note: Reference categories: ^a^ “preferred to retire at the time one expected to”; ^b^ “pulled toward early retirement”; ^c^ “no”; ^d^ “married/cohabiting man; ^e^ “high”; ^f^ “white-collar workers”; significant results are highlighted in **bold**; *** *p* ≤ 0.001.

## Data Availability

The data are not publicly available due to legal restrictions that guarantee the privacy of research participants. Overall statistics on group level (means, correlations, without demographics) are available on request from the corresponding author [MS-R].
